# Selection dramatically reduces effective population size in HIV-1 infection

**DOI:** 10.1186/1471-2148-8-133

**Published:** 2008-05-03

**Authors:** Yi Liu, John E Mittler

**Affiliations:** 1 Department of Microbiology, University of Washington School of Medicine, Seattle, Washington, 98195, USA

## Abstract

**Background:**

In HIV-1 evolution, a 100–100,000 fold discrepancy between census size and effective population size (*N*_*e*_) has been noted. Although it is well known that selection can reduce *N*_*e*_, high *in vivo *mutation and recombination rates complicate attempts to quantify the effects of selection on HIV-1 effective size.

**Results:**

We use the inbreeding coefficient and the variance in allele frequency at a linked neutral locus to estimate the reduction in *N*_*e *_due to selection in the presence of mutation and recombination. With biologically realistic mutation rates, the reduction in *N*_*e *_due to selection is determined by the strength of selection, i.e., the stronger the selection, the greater the reduction. However, the dependence of *N*_*e *_on selection can break down if recombination rates are very high (e.g., *r *≥ 0.1). With biologically likely recombination rates, our model suggests that recurrent selective sweeps similar to those observed *in vivo *can reduce within-host HIV-1 effective population sizes by a factor of 300 or more.

**Conclusion:**

Although other factors, such as unequal viral reproduction rates and limited migration between tissue compartments contribute to reductions in *N*_*e*_, our model suggests that recurrent selection plays a significant role in reducing HIV-1 effective population sizes *in vivo*.

## Background

The effective population size, *N*_*e*_, is defined as the size of an idealized population that has the same population genetics properties (generally those properties that measure the magnitude of random genetic drift) as the actual population. Most studies have estimated the within-host *N*_*e *_for HIV-1 during chronic infection to be ~10^3 ^[1-5], though one study estimated *N*_*e *_to be between 10^5 ^and 5 × 10^5 ^[6]. Even the highest of these estimates is about two orders of magnitude lower than the number of productively infected cells, estimated to be on the order of 10^7 ^to 10^8 ^cells [7]. Explanations for low *N*_*e *_values include unequal viral reproduction rates [2-5,8], structured populations [8-12], and recurrent selection [2-5,8]. The possibility that recurring selection may be reducing viral diversity is unsettling because most of the computational models used to estimate *N*_*e *_assume neutral evolution.

During a selective sweep of a favorable allele, any neutral alleles linked to the selected allele will rise in frequency and become overrepresented in the population. This process, called "hitchhiking", can reduce neutral diversity more than random genetic drift and therefore reduce *N*_*e *_[13]. Although selection has been acknowledged as a possible explanation for the low within-host effective population size during chronic HIV-1 infection [3,12], high mutation [14,15] and recombination rates [16-20] complicate attempts to study the effects of selection on HIV-1 *in vivo*. To address these issues, we extended a classic "inbreeding coefficient" method [21-23] to derive recurrence equations that account for the combined effects of selection, mutation, and recombination. We then used these equations to quantify the effects of selection on effective size using parameters relevant to HIV-1 evolution *in vivo*.

## Results and Discussion

### Overview of the genetic model

Our model follows the basic Wright-Fisher assumptions of a single haploid population of constant size with no subdivision or migration, non-overlapping generations, and random sampling of offspring each generation. We calculated *N*_*e *_in terms of the inbreeding effective size, which is based on the change of the average inbreeding coefficient (*F*) at a neutral locus (*L*) that is linked to a locus (*S*) that is under selection. The inbreeding coefficient is defined as the probability that two individuals are identical by descent (which means they are identical and have a common ancestor). Therefore, for the neutral locus *L*, two individuals are identical by descent if they are derived from a common ancestor and are identical at locus *L*, regardless of the status of locus *S*. Our approach to estimating *N*_*e *_was to determine changes in the inbreeding coefficient at the neutral locus in the presence and absence of selection and recombination. The effective population size was defined as the size of the neutral population that gave changes in the inbreeding coefficient that were equal to those observed in the presence of selection and recombination.

**Figure 1:**
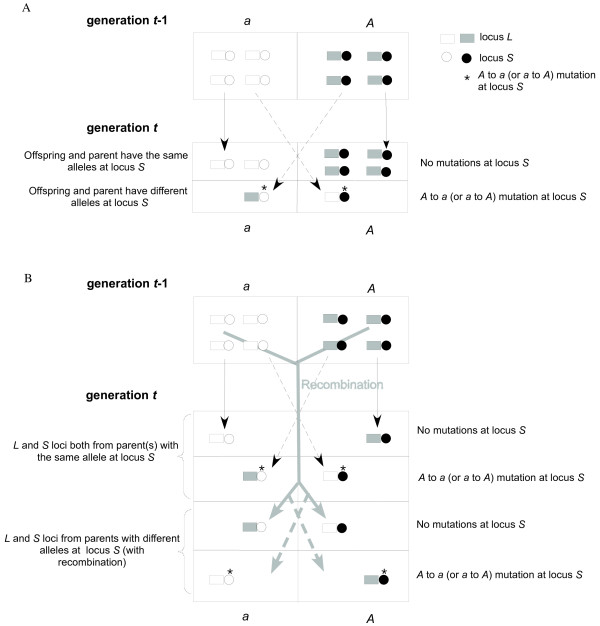
**Illustration of the genetic model**. A) In the absence of recombination, an offspring with allele *a *can be derived from a parent with allele *a *(without mutation) or a parent with allele *A *(with an *A *to *a *mutation); an offspring with allele *A *can be derived from a parent with allele *A *(without mutation) or a parent with allele *a *(with an *a *to *A *mutation). B) In the presence of recombination, an offspring with allele *a *at locus *S *can be derived from parent(s) in the previous generation by four pathways: 1) Locus *S *from a parent with allele *a *without mutation or recombination, (or with recombination between another parent with allele *a*). 2) Locus *S *from a parent with allele *A *following an *A *to *a *mutation but no recombination (or with recombination between another parent with allele *A*). 3) Locus *S *from a parent with allele *a *without mutation, but with recombination between another parent with allele *A*. 4) Locus *S *from a parent with allele *A *following an *A *to *a *mutation and recombination between another parent with allele *a*. An offspring with allele *A *can be derived from parent(s) in four pathways similar to those described above. For the purpose of illustration, only 8 genomes were presented in generation *t*-1 and *t*.

As shown in Figure 1A, in the absence of recombination, an offspring can be derived from a parent in the previous generation with either allele *a *or *A *at locus *S*. An offspring with allele *a *can be derived by two pathways: from a parent with allele *a *(without mutation) or a parent with allele *A *(with an *A *to *a *mutation). An offspring with allele *A *can be derived by two similar pathways. Therefore, *F*_*t *_(the value of *F *at time *t*) will be the sum of the probability that two offspring are derived from a certain combination of parents (both with allele *A*, both with allele *a*, and one with allele *A *and the other with allele *a*) times the probability that the offspring are identical by descent at locus *L *(see Appendix).

In the presence of recombination, loci *L *and *S *can be derived from different parents (Figure 1B). An offspring with allele *a *or *A *at locus *S *can be derived from one or more parents in the previous generation by the four pathways illustrated in Figure 1B. As above, *F*_*t *_will be the sum of the probability that the two offspring are derived from a certain combination of pathways (both having locus *S *from parents with allele *A*, both having locus *S *from parents with allele *a*, one having locus *S *from a parent with allele *a *and the other having locus *S *from a parent with allele *A*) times the probability that the offspring derived from these pathways are identical by descent at locus *L *(see APPENDIX).

### Effect of selection on effective population size

We used the ratio *N/N*_*e *_to summarize the reduction in *N*_*e *_due to selection from the start of selection at *t *= 0 until *t *= *t*_*nearlyfixed *_[the time when the frequency of the advantageous allele reaches (*N*-1)/*N*]. This last approximation is helpful because fixation time is asymptotic, with the advantageous allele never reaching 100% in a deterministic model.

**Figure 2:**
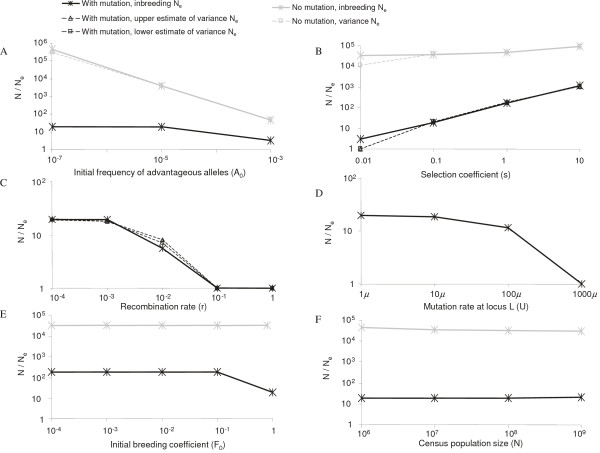
**Reduction in *N*_*e *_following a selective replacement with and without mutations in loci *L *and *S***. A) The effect of different initial frequencies of the advantageous allele. B) The effect of different selection coefficients. C) The effect of different recombination rates. D) The effect of different mutation rates at locus *L*. E) The effect of different initial inbreeding coefficients. F) The effect of different census population sizes. Panels A and C-F all assume *s *= 0.1. Solid lines indicate that the *N*/*N*_*e *_ratios are based on the inbreeding coefficient *F*_*t*_; dashed lines indicate that the *N*/*N*_*e *_ratios are based on the variance effective population sizes estimated from our simulations. In the presence of mutation, the dashed lines indicate the *N*/*N*_*e *_ratios based on the upper and lower estimates of variance effective populations size. Black lines indicate cases with mutation; grey lines indicate cases without mutation. Unless otherwise specified, the following parameters were used: in the absence of mutation, *μ *= 0, *v *= *0*, *U *= 0, *N *= 10^7^, *s *= 0.1, *A*_0 _= 10^-6^, *r *= 0, and *F*_0 _= *F*_*AA*,0 _= *F*_*aa*,0 _= *F*_*Aa*,0 _= 0.1; in the presence of mutation, *μ *= 2.5 × 10^-5^, *v *= *μ*/3, *U *= *μ*, *N *= 10^7^, *s *= 0.1, *A*_0 _= 0, *r *= 0, and *F*_0 _= *F*_*aa*,0 _= 1, *F*_*AA*,0 _= *F*_*Aa*,0 _= 0.

In the absence of mutation, the reduction in *N*_*e *_due to selection was most strongly affected by the initial frequency of the advantageous allele, *A*_0 _(Figure 2A). In the presence of mutation, the reduction in *N*_*e *_due to selection was most sensitive to the selective advantage, *s*, of the advantageous allele (Figure 2B). Indeed, for a homogeneous population of *N *= 10^7^, the *N*/*N*_*e *_ratio increased 6 – 9 fold with each 10-fold increase in the selection coefficient in the presence of mutation. However, recombination can break the hitchhiking effect of selection on *N*_*e *_(Figure 2C). For example, when *r *≤ 10^-3^, for locus *L *with *U *= *μ*, selection with *s *= 0.1 reduced *N*_*e *_by ~20 fold. In contrast, when *r *≥ 0.1, selection with *s *= 0.1 had little effect on *N*_*e*_.

Effective population sizes calculated from the inbreeding coefficient (inbreeding *N*_*e*_) are usually the same as those calculated from the variance in the allele frequency (variance *N*_*e*_), though exceptions do occur [24-27]. To validate our results, we estimated the effect of selection on *N*_*e *_by calculating the variance in the frequency of the linked neutral allele from simulations using the same genetic model. Values for the inbreeding *N*_*e *_obtained from the calculations above were generally consistent with the estimates of the variance *N*_*e *_derived from these simulations (Figure 2A to 2C). We noted that there was an approximate 3-fold difference in the *N*_*e *_values between the two methods when *s *= 0.01 (Figure 2B). This is likely due to the fact that the inbreeding *N*_*e *_was estimated using a strict deterministic model; while the variance *N*_*e *_was estimated from simulations of *s *= 0.01, where genetic drift plays a bigger role.

A very high mutation rate at the neutral locus *L *(e.g., *U *= 1000*μ*) also diminished the reduction in *N*_*e *_due to selection (Figure 2D). In the absence of mutation, the effect of selection was insensitive to changes in the initial homogeneity at locus *L *(Figure 2E). In the presence of mutation, selection with an initially heterogeneous population at locus *L *caused greater reductions in *N*_*e *_than selection with an initially homogeneous population. For *F*_0 _less than 0.1, however, further increases in the initial heterogeneity (i.e., making *F*_0 _even lower) did not lead to further reductions in *N*_*e *_through selection. Interestingly, reductions in *N*_*e*_/*N *due to selection were insensitive to changes in the census population size, *N *(Figure 2F).

### Effect of recurrent selection on effective population size

**Figure 3:**
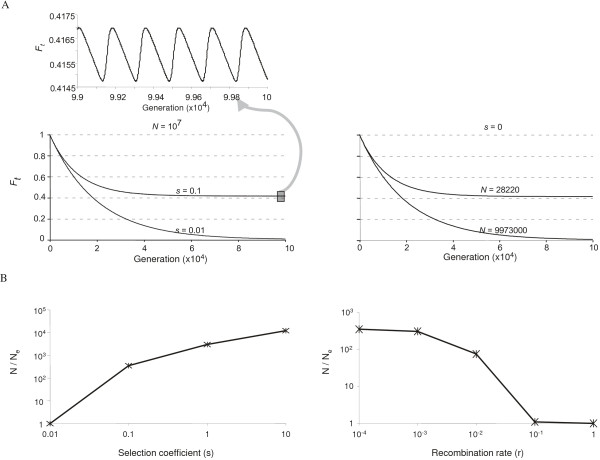
**Reduction in *N*_*e *_due to recurrent selection**. A) The changes of *F*_*t *_over time under selection (left panels) and under neutrality (right panel), in the absence of recombination. B) The effects of different selection coefficients (left panel, *r *= 0) and different recombination rates (right panel, *s *= 0.1). The starting parameters were: *F*_0 _= *F*_*aa*,0 _= 1, *F*_*AA*,0 _= *F*_*Aa*,0 _= 0, *A*_0 _= 0, *U = μ = *2.5 × 10^-5^, *v *= *μ*/3.

For a homogeneous population under recurrent selection, the inbreeding coefficient of the neutral allele decreased until it reached a quasi-steady state, where it fluctuated in a regular "sawtooth" fashion (Figure 3A). The effect of recurrent selection on *N*_*e *_was sensitive to selection strength. For example, for a homogeneous population of *N *= 10^7 ^and *U *= *μ*, the decline of *F*_*t *_over time under recurrent selection with *s *= 0.01 overlapped the neutral curve when *N *= 9,973,000, while the decline of *F*_*t *_under recurrent selection with *s *= 0.1 overlapped the neutral curve when *N *= 28,220 (Figure 3A). In other words, recurrent selection had little effect on *N*_*e *_when *s *= 0.01, while recurrent selection reduced *N*_*e *_by over 300-fold when *s *= 0.1 (Figure 3A and 3B). This reduction in *N*_*e *_by recurrent selection could be diminished by high recombination rates (Figure 3B). Although recombination had little impact on the reduction in *N*_*e *_due to selection under a model with *s *= 0.1 and *r *≤ 10^-3^, with *r *≥ 0.1, recombination completely broke the hitchhiking effects of selection on *N*_*e *_with *s *= 0.1.

## Conclusion

We examined the combined effects of selection, mutation, and recombination on the effective population size of a neutral locus that is linked to a locus under selection. Consistent with other studies [21-23], we found that selection can increase the inbreeding coefficient and reduce the inbreeding effective population size. Without mutation, this reduction is primarily determined by the initial frequency of the advantageous allele, i.e., the lower the initial frequency, the greater the effect. With mutation, this reduction is mostly determined by the strength of selection, i.e., the stronger the selection, the greater the effect. With moderate recombination rates (e.g., *r *≤ 10^-3^), recurrent selection can substantially lower *N*_*e*_, though the hitchhiking effect disappears if the recombination rates are very high (e.g., *r *≥ 0.1).

The effective population size of HIV-1 during chronic infection has been shown to be 100- to 100,000-fold lower than within-host census size. Indeed, CTL responses are a driving force of HIV-1 evolution and these responses continuously select for escape mutants during chronic infection [28-30]. In a comprehensive study of viral evolution and CTL responses during the first four years of HIV-1 infection in one subject, Liu *et al*. [30,31] found that of the 25 epitopes detected in this subject, 17 were largely replaced by mutants over time. The selection coefficients for the CTL escape mutant(s) of a single epitope ranged from 0.2 to 0.4 during acute infection and from 0.0024 to 0.15 during chronic infection, with an average of 0.03 [30]. Humoral and escape-specific CTL responses impose additional selective pressures not quantified in Liu *et al*. [30,31]. With low to moderate recombination rates, our model shows that recurrent selection with *s *= 0.1 reduces viral effective population size by approximately 300-fold. Therefore, during HIV-1 infection, selection alone is likely to reduce the viral effective population size to an *N*_*e *_of ~10^5^. This result is close to the estimate of *N*_*e*_~5 × 10^5 ^that Rouzine and Coffin [6] obtained from a model that accounts for selection. The small discrepancy may be due to their use of a lower mutation rate (10^-5 ^vs. 2.5 × 10^-5 ^in our study) and possible biased sampling of sites with higher underlying mutation rates in their study [5].

With high recombination rates, our model predicts that selection has little effect on *N*_*e*_. Observations of 3 to 13 cross-over events per virion *in vitro *[17-20] suggest an intrinsic recombination rate of 10^-4 ^to 10^-3 ^per adjacent site per generation. However, this range is not relevant to our model since these estimates were obtained using heterozygous virions, which may not be abundant *in vivo*. While Jung and colleagues [32] have demonstrated that cells in the spleen are infected with multiple viruses (a pre-requisite for the formation of heterozygous virions), they did not determine how often heterozygous virions are formed. More relevant is data in which SCID-HU mice were infected with a 50:50 mixture of two marked strains [19]. Two-to-three weeks after infection, an average of ~0.01% of infected cells carried a phenotypic marker of recombination (present on half of all recombinants). Conservatively assuming a single generation of recombination, we estimate from equation (11, Appendix) that the probability of recombination between their two markers (which were 408 bp apart) was *r = ~ p*_*Aa*_/(*p*_*A*_*p*_*a*_) = 0.0001/(0.5 × 0.5) = ~4 × 10^-4 ^per virion per generation – a value too low to break the hitchhiking effects of selection in our model. However, we recognize these are approximate values obtained from a somewhat artificial system. HIV-1 evolution studies could benefit from additional studies of marked viruses in animal models and clever retrospective analyses of *in vivo *data from humans to determine evolutionarily relevant recombination rates.

## Methods

### Genetic model

We assume a Wright-Fisher model with a neutral locus *L *that is linked to a locus under selection, locus *S*. The selected locus has two alleles, an advantageous allele, *A*, with a fitness *w *= 1 + *s*, and a disadvantageous allele, *a*, with a fitness of 1. Allele *A *mutates to *a *at rate *μ *and allele *a *mutates to *A *at rate *ν*, while neutral mutations at locus *L *occur at rate *U*. A description of all the characters, parameters, and variables used in this study is listed in Table 1. For the purposes of calculation, we assume the following parameters are known: the initial frequency of allele *A *(*A*_0_); the initial frequency of allele *a *(*a*_0_); and the initial inbreeding coefficient at locus *L *among all individuals (*F*_0_), among individuals with allele *A *(*F*_*AA*,0_), among individuals with allele *a *(*F*_*aa*,0_), and between individuals with allele *A *and those with allele *a *(*F*_*Aa*,0_).

**Table 1:** Description of characters, parameters and variables.

Characters	Description
*S*	Locus under selection.
*L*	Neutral locus linked to locus *S*.
*A*	Advantageous allele at locus *S*.
*a*	Disadvantageous allele at locus *S*.

Parameters	

*N*	Census population size.
*s*	Selection coefficient.
*W*	Fitness of the advantageous allele *A*, *w *= 1+*s*.
*μ*	Probability that locus *S *mutates from *A *to *a *per virion per generation.
*ν*	Probability that locus *S *mutates from *a *to *A *per virion per generation.
*U*	Probability that mutation occurs at locus *L *per virion per generation.
*r*	Probability of recombination between loci *L *and *S *per virion per generation.

Variables	

*N*_*e*_	Effective population size.
*A*_*t*_	Frequency of allele *A *at locus *S *at generation *t*.
*a*_*t*_	Frequency of allele *a *at locus *S *at generation *t*.
*F*_*t*_	Probability that two alleles at locus *L *are identical by descent at generation *t *(equivalent to the inbreeding coefficient in classic population genetics).
$\hat{F}$	Inbreeding coefficient at equilibrium.
*F*_*AA*, *t*_	*F *of locus *L *between offspring with allele *A*, at generation *t*.
*F*_*aa*, *t*_	*F *of locus *L *between offspring with allele *a*, at generation *t*.
*F*_*Aa*, *t*_	*F *of locus *L *between offspring with alleles *A *and *a*, at generation *t*.
*p*_*A*, *t*_	Probability that an offspring at generation *t *is derived from a parent with allele *A *at generation *t*-1.
*p*_*a*, *t*_	Probability that an offspring at generation *t *is derived from a parent with allele *a *at generation *t*-1.
*p*_*A*→*A*, *t*_	Probability that an offspring at generation *t *is derived from a parent with allele *A*, given that the offspring has allele *A*.
*p*_*A*→*a*, *t*_	Probability that an offspring at generation *t *is derived from a parent with allele *A*, given that the offspring has allele *a*.
*p*_*a*→*a*, *t*_	Probability that an offspring at generation *t *is derived from a parent with allele *a*, given that the offspring has allele *a*.
*p*_*a*→*A*, *t*_	Probability that an offspring at generation *t *is derived from a parent with allele *a*, given that the offspring has allele *A*.
*P*_*AA*, *t*_	Probability of an individual at generation *t *having alleles at loci *L *and *S *both being derived from individual(s) with allele *A *at locus *S*.
*P*_*Aa*, *t*_	Probability of an individual at generation *t *having locus *L *derived from an individual with allele *A *at locus *S *and locus *S *derived from an individual with allele *a *at locus *S*.
*P*_*aa*, *t*_	Probability of an individual at generation *t *having alleles at loci *L *and *S *both being derived from individual(s) with allele *a *at locus *S*.
*P*_*aA*, *t*_	Probability of an individual at generation *t *having locus *L *derived from an individual with allele *a *at locus *S *and locus *S *derived from an individual with allele *A *at locus *S*.

### Parameters for HIV-1

The average mutation rate of HIV-1 has been estimated to be 2.5 × 10^-5 ^per nucleotide per generation [14], although one recent study estimated a higher mutation rate of ~8.5 × 10^-5 ^per site per generation [15]. Assuming that any nucleotide substitution at a defined nucleotide site shifts locus *S *from the advantageous to the disadvantageous state, we defined *μ *= 2.5 × 10^-5 ^per generation. Assuming that only a particular nucleotide substitution at this site increases fitness, we set *ν *= *μ*/3. Since the census sizes of productively HIV-1 infected cells *in vivo *exceeds 10^7 ^[7,33], most of the comparisons in this study were with *N *= 10^7^. Since the accumulation of advantageous alleles in populations is more stochastic as *N *decreases, we only examined populations with *N *≥ 10^6^.

### Effect of selection on effective population size without mutation

Under selection, the inbreeding coefficient of the linked neutral locus will increase faster than expected by random genetic drift until the selected advantageous allele is fixed (*A*_*t *_= 100%). Because we are using a deterministic model, fixation time is asymptotic. To quantify the effect of selection, we determined the average time for an advantageous allele to approach fixation, *t*_*nearlyfixed*_, and the value of *F *at *t*_*nearlyfixed*_. *t*_*nearlyfixed *_can be calculated from $t=\log(\frac{A_{t}a_{0}}{a_{t}A_{0}})/\log(w)$, where *t *is the time just before the favored allele *A *at locus *S *becomes fixed; i.e., when $A_{t}=\frac{N-1}{N}$ and $a_{t}=\frac{1}{N}$. *F *was calculated with *μ *= 0, *v *= 0, and *U *= 0. The corresponding *N*_*e *_is defined here as the population size under neutrality that will increase *F *from *F*_0 _to $F_{t_{nearlyfixed}}$ between *t *= 0 and *t *= *t*_*nearlyfixed*_. We determined the corresponding *N*_*e *_under the following conditions: *N *= 10^6 ^to 10^9^; *s *= 0.01 to 10; *A*_0 _= 10^-7 ^to 10^-3^; and *F*_0 _= *F*_*AA*,0 _= *F*_*aa*,0 _= *F*_*Aa*,0 _= 10^-4 ^to 0.8 (if *F*_0 _= 1, *F *will not change over time without mutation, regardless of selection).

### Effect of selection on effective population size with mutation and recombination

The frequency of the *A *allele cannot be maintained at 100% with the occurrence of the back mutation from *A *to *a *at locus *S*. Therefore *t*_*nearlyfixed *_was set to the time that *A*_*t *_and *a*_*t *_reached equilibrium, i.e., when *A*_*t *_= *A*_*t*+1_. The corresponding *N*_*e*_, the population size under neutrality that will increase *F *from *F*_0 _to $F_{t_{nearlyfixed}}$ between *t *= 0 and *t *= *t*_*nearlyfixed*_, was determined using numerical iteration [Appendix equation (2)]. We determined the corresponding *N*_*e *_under the following conditions: *N *= 10^6 ^to 10^9^; *s *= 0.01 to 10; *A*_0 _= 0 to 10^-3^; *F*_0 _= *F*_*aa*,0 _= 10^-4 ^to 1; *F*_*AA*,0 _= *F*_*Aa*,0 _= 0, if *A*_0 _= 0 and *F*_*AA*,0 _= *F*_*Aa*,0 _= *F*_0_, if *A*_0 _> 0; *μ *= 2.5 × 10^-5^, *v *= *μ*/3, *U *= *μ *to 1000*μ*, and *r *= 0 to 1. With these high advantageous mutation rates and large population sizes (*Nv *>> 1), individuals with allele *a *had mutations to allele *A *in almost every generation, preventing advantageous allele *A *from being lost from the population due to genetic drift.

### Effect of recurrent selection on effective population size

With the fixation of the advantageous allele *A*, the inbreeding coefficient of locus *L *will undergo a nearly neutral change unless new alleles linked to locus *L *become advantageous. To estimate the effect of recurrent selection on *N*_*e*_, we assumed that all loci under selection are linked to locus *L *in the absence of recombination. We also assumed that each selected locus was under sequential selection, i.e., when the frequency of an advantageous allele reached 99.9% at generation *t*, we assumed that another locus started to undergo selection (calculated by setting *A*_*t *_= 0, *F*_*aa*, *t *_= *F*_*t*_, *F*_*AA*, *t *_= 0, and *F*_*Aa*, *t *_= 0). For simplicity, we assumed that all of the selected loci have the same mutation rate and selection coefficient. We calculated *F *under recurrent selection under the following conditions: *N *= 10^7^, *A*_0 _= 0, *F*_*AA*,0 _= *F*_*Aa*,0 _= 0, *F*_0 _= *F*_*aa*,0 _= 1, *μ *= 2.5 × 10^-5^, *v *= *μ*/3, and *U *= *μ*; *s *= 0.01 to 10; and *r *= 0 to 1.

### Estimate of the effect of selection on variance effective population size by simulation

The change in the average inbreeding coefficient is one of several criteria used to estimate effective population size [24-27]. To validate our results using a different measure of effective population size, we estimated the effect of selection on *N*_*e *_by calculating the variance in the frequency of the linked neutral allele from simulations using the genetic model described above. The parameters used in these simulations were the same as those used for the calculation for the inbreeding coefficient described above. When simulating selection in the absence of mutation, the simulations were performed under the following conditions: *N *= 10^7^; *s *= 0.01 to 10; *A*_0 _= 10^-7 ^to 10^-3^; *F*_0 _= *F*_*AA*,0 _= *F*_*aa*,0 _= *F*_*Aa*,0 _= 0.1; *μ *= *v *= *U *= *0; *and *r *= 0. When simulating selection in the presence of mutation, the simulations were performed with the following conditions: *N *= 10^7^; *s *= 0.01 to 10; *A*_0 _= 0; *F*_0 _= *F*_*aa*,0 _= 1, *F*_*AA*,0 _= *F*_*Aa*,0 _= 0; *μ *= 2.5 × 10^-5^, *v *= *μ*/3, and *U *= *μ*; *s *= 0.01 to 10; and *r *= 0 to 1. Since the deterministic model assumes an infinite population size, we only examined a large population size of 10^7^. For each condition, 100,000 simulations were repeated. We calculated the variance of the allele frequency at the linked neutral locus *L *at the corresponding *t*_*nearlyfixed*_. Under neutrality in the absence of mutation, the allele frequency variance can be calculated by $p(1-p)[1-{\left(1-\frac{1}{N}\right)}^{t}]$[34]. Therefore, the population size under neutrality (*N*_*e*_) that has the same variance in allele frequency as the population under selection can be determined using numerical iteration. In the presence of mutation, we used simulation to determine the range of the population size under neutrality. These were used to determine the range of allele frequency variances that matched the frequency variance under selection at the corresponding *t*_*nearlyfixed*_.

## Authors' contributions

YL and JM jointly conceived the study. YL derived the equations, wrote the computer code, performed the computational experiments, and drafted the manuscript. JM advised on the study design, participated in the analysis of the mathematical and computational data, and helped draft the manuscript. Both authors have read and approved the paper.

## Appendix

### Recurrence equation for *F *in the absence of selection

In the absence of mutation or selection, the inbreeding coefficient is

(1) $$F_{t}=\frac{1}{N}+(1-\frac{1}{N})F_{t-1}=1-{\left(1-\frac{1}{N}\right)}^{t}(1-F_{0})$$

where *t *is time in generations and *N *is the population size [26]. $\frac{1}{N}$ gives the probability that two offspring are derived from same parent in which case the probability of them being identical by descendent is 1. $\left(1-\frac{1}{N}\right)$ is the probability that two offspring are derived from different parents in which case the probability of them being identical by descendent is *F*_*t*-1_. In the presence of mutation, $F_{t}=[\frac{1}{N}+(1-\frac{1}{N})F_{t-1}]{\left(1-U\right)}^{2}$[35]. To obtain *F*_*t *_in terms of *F*_0_, let $\alpha =\frac{1}{N}\times {\left(1-U\right)}^{2}$, and $\beta =(1-\frac{1}{N})\times {\left(1-U\right)}^{2}$. This gives

*F*_1 _= *α *+
*βF*_0_

*F*_2 _= *α *+
*βF*_1 _= *α *+ *β *×
(*α *+ *βF*_0_) = *α *+
*αβ *+
*β*^2^*F*_0_

*F*_3 _= *α *+
*βF*_2 _= *α *+ *β *×
(*α *+ *αβ *+
*β*^2^*F*_0_) = *α *+
*αβ *+ *αβ*^2 ^+
*β*^3^*F*_0_

*F*_*t *_= *α *+
*αβ *+ *αβ*^2 ^+
*αβ*^3 ^+ ... +
*αβ*_*t*-1 _+
*β*^*t*^*F*_0_.

The formula, 1 + *x *+ ... + *x*^*n*-1 ^= (1-*x*^*n*^)/(1-*x*), gives the following:

(2) *F*_*t *_= *α *(1 -
*β*^*t*^)/(1 - *β*) +
*β*^*t*^*F*_0_,

As *t *approaches infinity, *F *converges to the equilibrium $\hat{F}=\frac{\alpha }{\left(1-\beta \right)}\approx \frac{1}{1+2NU}$, as shown previously by Kimura and Crow [35].

### Recurrence equations for *F *in the presence of a selected locus without recombination

In the presence of selection, the *F *value at time *t *is the sum of the probability of two offspring being derived from parents having alleles *AA*, *aa*, or *Aa *at locus *S *multiplied by the probability that the offspring will be identical by descent at locus *L*. In other words:

(3) $$F_{t}=\left\{p_{A,t}^{2}[\frac{1}{NA_{t-1}}+(1-\frac{1}{NA_{t-1}})F_{AA,t-1}]+p_{a,t}^{2}[\frac{1}{Na_{t-1}}+(1-\frac{1}{Na_{t-1}})F_{aa,t-1}]+2p_{A,t}p_{a,t}F_{Aa,t-1}\right\}{\left(1-U\right)}^{2}.$$

Here, *A*_*t*-1 _and *a*_*t*-1 _are the frequencies of the advantageous and disadvantageous alleles at locus *S *at generation *t*-1. $p_{A,t}=\frac{wA_{t-1}}{wA_{t-1}+a_{t-1}}$ and $p_{a,t}=\frac{a_{t-1}}{wA_{t-1}+a_{t-1}}$ give the probabilities that an offspring at generation *t *is derived from a parent at generation *t*-1 with allele *A *or *a*, respectively. *F*_*AA*_, *F*_*Aa*_, and *F*_*aa *_give the probabilities that parents with the indicated alleles will be identical by descent at locus *L*. Given that both parents have allele *A *or *a *at locus *S*, the $\frac{1}{NA_{t-1}}$ and $\frac{1}{Na_{t-1}}$ terms respectively give the probabilities that two offspring have the same parent (in which case the probability of being identical by descent at locus *L*, in the absence of mutation is 1). The $1-\frac{1}{NA_{t-1}}$ and $1-\frac{1}{Na_{t-1}}$ terms give the probability that the two offspring came from different parents (in which case the probabilities of identity by descent at locus *L*, in the absence of mutation, are *F*_*AA*, *t*-1 _and *F*_*aa*, *t*-1 _respectively). The term (1-*U*)^2 ^accounts for the fact that two individuals cannot be identical by descent if there is a mutation at the neutral locus *L*.

If the parameters *w*, *μ*, *v*, *U*, *A*_0_, *a*_0_, *F*_0_, *F*_*AA*,0_, *F*_*aa*,0_, and *F*_*Aa*,0 _are known, *F*_1 _can be calculated using equation (3). In addition, *F*_*AA*,1_, *F*_*aa*,1_, and *F*_*Aa*,1 _can be calculated using the following equations:

(4) $$\begin{array}{l} F_{AA,t}=\{{\left(p_{A\to A,t}\right)}^{2}[\frac{1}{NA_{t-1}}+(1-\frac{1}{NA_{t-1}})F_{AA,t-1}]+{\left(p_{a\to A,t}\right)}^{2}[\frac{1}{Na_{t-1}}+(1-\frac{1}{Na_{t-1}})F_{aa,t-1}]\\ +2p_{A\to A,t}p_{a\to A,t}F_{Aa,t-1}\}{\left(1-U\right)}^{2}, \end{array}$$

(5) $$\begin{array}{l} F_{aa,t}=\{{\left(p_{a\to a,t}\right)}^{2}[\frac{1}{Na_{t-1}}+(1-\frac{1}{Na_{t-1}})F_{aa,t-1}]+{\left(p_{A\to a,t}\right)}^{2}[\frac{1}{NA_{t-1}}+(1-\frac{1}{NA_{t-1}})F_{AA,t-1}]\\ +2p_{A\to a,t}p_{a\to a,t}F_{Aa,t-1}\}{\left(1-U\right)}^{2} \end{array}$$

and

(6) *F*_*Aa*,*t *_=
(*p*_*A*→*A*,*t
*_*p*_*a*→*a*,*t
*_*F*_*Aa*,*t*-1 _+
*p*_*a*→*a*,*t
*_*p*_*a*→*A*,*t
*_*F*_*aa*,*t*-1 _+
*p*_*A*→*a*,*t
*_*p*_*A*→*A*,*t
*_*F*_*AA*,*t*-1 _+
*p*_*A*→*a*,*t
*_*p*_*a*→*A*,*t
*_*F*_*Aa*,*t*-1_)(1 -
*U*)^2^

Where *p*_*x*→*y*, *t *_is the probability that a sampled offspring is descended from a parent with allele *x *given that the offspring has allele *y *at locus *S*. The reasoning behind equations (4) – (6) is similar to that for equation (3). For each offspring, *p*_*x*→*y*, *t *_can be calculated as the probability of the parent having allele *x *at locus *S *multiplied by the probability that *x *mutates to *y *(or fails to mutate, if *x *= *y*), divided by the probability that the offspring is *y*. In other words,

$$\begin{array}{cc} p_{A\to A,t}=\frac{w\times A_{t-1}}{w\times A_{t-1}+a_{t-1}}(1-\mu )/A_{t}, & p_{a\to A,t}=\frac{a_{t-1}}{w\times A_{t-1}+a_{t-1}}v/A_{t} \end{array}$$

and

$$\begin{array}{cc} p_{a\to a,t}=\frac{a_{t-1}}{w\times A_{t-1}+a_{t-1}}(1-v)/a_{t}, & p_{A\to a,t}=\frac{w\times A_{t-1}}{w\times A_{t-1}+a_{t-1}}\mu /a_{t} \end{array}$$

where *A*_*t *_and *a*_*t *_are given by

(7) $$A_{t}=\frac{w\times A_{t-1}}{w\times A_{t-1}+a_{t-1}}(1-\mu )+\frac{a_{t-1}}{w\times A_{t-1}+a_{t-1}}v$$

(8) $$a_{t}=\frac{a_{t-1}}{w\times A_{t-1}+a_{t-1}}(1-v)+\frac{wA_{t-1}}{w\times A_{t-1}+a_{t-1}}\mu$$

When *A*_1_, *a*_1_, *F*_1_, *F*_*AA*,1_, *F*_*aa*,1_, and *F*_*Aa*,1 _are available, we can calculate *F*_2 _using equation (3), and *F*_*AA*,2_, *F*_*aa*,2_, and *F*_*Aa*,2 _using equations (4) to (6). Therefore, *F*_*t *_can be obtained by iteration.

### Recurrence equations for *F *in the presence of a selected locus and recombination

Assuming that loci *L *and *S *recombine with a probability *r *per generation, we obtain

(9) $$P_{AA,t}=p_{A,t}(1-r)+p_{A,t}r\frac{Np_{A,t}-1}{N-1}$$

(10) $$P_{aa,t}=p_{a,t}(1-r)+p_{a,t}r\frac{Np_{a,t}-1}{N-1}$$

(11) $$P_{Aa,t}=p_{aA,t}=p_{A,t}r\frac{Np_{a,t}}{N-1}$$

where *p*_*xy*, *t *_is the probability that an individual at generation *t *has a neutral locus *L *derived from an individual with allele *x *at locus *S*, and a selected locus *S *derived from an individual with allele *y *at locus *S *(*x *and *y *can be *A *or *a*). This probability is the sum of the probability of no recombination and the probability of recombination between individuals with indicated allele at locus *S*. The -1's in the (*Np*_*x*, *t *_- 1) and (*N *- 1) terms above account for the fact that a haploid individual cannot recombine with itself.

Similar to equations (3) – (6), with recombination,

(12) $$\begin{array}{l} F_{t}=\left\{\begin{array}{l} \left(p_{AA,t}^{2}+p_{Aa,t}^{2}+2p_{AA,t}p_{Aa,t})[\frac{1}{NA_{t-1}}+(1-\frac{1}{NA_{t-1}})F_{AA,t-1}\right]\\ +(p_{aA,t}^{2}+p_{aa,t}^{2}+2p_{aA,t}p_{aa,t})[\frac{1}{Na_{t-1}}+(1-\frac{1}{Na_{t-1}})F_{aa,t-1}]\\ +2(p_{AA,t}p_{aA,t}+p_{AA,t}p_{aa,t}+p_{aA,t}p_{Aa,t}+p_{aa,t}p_{Aa,t})F_{Aa,t-1} \end{array}\right\}{\left(1-U\right)}^{2}\\ =\left\{p_{A,t}^{2}[\frac{1}{NA_{t-1}}+(1-\frac{1}{NA_{t-1}})F_{AA,t-1}]+p_{a,t}^{2}[\frac{1}{Na_{t-1}}+(1-\frac{1}{Na_{t-1}})F_{aa,t-1}]+2p_{A,t}p_{a,t}F_{Aa,t-1}\right\}{\left(1-U\right)}^{2} \end{array}$$

(13) $$\begin{array}{l} F_{AA,t}=\{{\left(\frac{p_{AA}(1-\mu )+p_{Aa}v}{A_{t}}\right)}^{2}[\frac{1}{NA_{t-1}}+\text{(}1-\frac{1}{NA_{t-1}}\text{)}F_{AA\text{, }t-1}\text{]}\\ {+(\frac{p_{aA}(1-\mu )+p_{aa}v}{A_{t}})}^{\text{2}}[\frac{1}{Na_{t-1}}+(1-\frac{1}{Na_{t-1}})F_{aa\text{, }t-1}]\\ +\frac{2[p_{AA}(1-\mu )\times p_{aA}(1-\mu )+p_{AA}(1-\mu )\times p_{aa}v+p_{aA}(1-\mu )\times p_{Aa}v+p_{Aa}v\times p_{aa}v]}{A_{t}^{2}}F_{Aa,t-1}\}\text{​}{\left(1-U\right)}^{2} \end{array}$$

(14) $$\begin{array}{l} F_{aa,t}=\{{\left(\frac{p_{Aa}(1-v)+p_{AA}\mu }{a_{t}}\right)}^{2}[\frac{1}{NA_{t-1}}+(1-\frac{1}{NA_{t-1}})F_{AA\text{, }t-1}]\\ {+(\frac{p_{aa}(1-v)+p_{aA}\mu }{a_{t}})}^{2}[\frac{1}{Na_{t-1}}+(1-\frac{1}{Na_{t-1}})F_{aa\text{, }t-1}]\\ +\frac{2[p_{Aa}(1-v)\times p_{aa}(1-v)+p_{Aa}(1-v)\times p_{aA}\mu +p_{aa}(1-v)\times p_{AA}\mu +p_{AA}\mu \times p_{aA}\mu ]}{a_{t}^{2}}F_{Aa,t-1}\}\text{​}{\left(1-U\right)}^{2} \end{array}$$

(15) $$\begin{array}{l} F_{Aa,t}=\{(\frac{p_{AA}(1-\mu )+p_{Aa}v}{A_{t}})\times (\frac{p_{Aa}(1-v)+p_{AA}\mu }{a_{t}})[\frac{1}{NA_{t-1}}+\text{(}1-\frac{1}{NA_{t-1}}\text{)}F_{AA\text{, }t-1}\text{]}\\ \text{+(}\frac{p_{aA}(1-\mu )+p_{aa}v}{A_{t}}\text{)}\times \text{(}\frac{p_{aa}(1-v)+p_{aA}\mu }{a_{t}}\text{)[}\frac{1}{Na_{t-1}}+\text{(}1-\frac{1}{Na_{t-1}}\text{)}F_{aa\text{, }t-1}\text{]}\\ \text{+}\frac{2p_{AA}p_{aA}\mu (1-\mu )+2p_{Aa}p_{aa}v(1-v)+(p_{AA}p_{aa}+p_{aA}p_{Aa})[(1-\mu )(1-v)+\mu v]}{A_{t}a_{t}}F_{Aa\text{, }t-1}\text{\}}{\left(1-U\right)}^{2} \end{array}.$$

where *A*_*t *_and *a*_*t *_are calculated using equations (7) and (8).
